# Estimation of auditory steady-state responses based on the averaging of independent EEG epochs

**DOI:** 10.1371/journal.pone.0206018

**Published:** 2019-01-24

**Authors:** Pavel Prado-Gutierrez, Eduardo Martínez-Montes, Alejandro Weinstein, Matías Zañartu

**Affiliations:** 1 Advanced Center for Electrical and Electronic Engineering, Universidad Técnica Federico Santa María, Valparaíso, Chile; 2 Neuroinformatics Department, Cuban Neuroscience Center, Havana, Cuba; 3 Biomedical Engineering School, Universidad de Valparaíso, Valparaíso, Chile; 4 Department of Electronic Engineering, Universidad Técnica Federico Santa María, Valparaíso, Chile; Universidad de Salamanca, SPAIN

## Abstract

The amplitude of auditory steady-state responses (ASSRs) generated in the brainstem of rats exponentially decreases over the sequential averaging of EEG epochs. This behavior is partially due to the adaptation of the ASSR induced by the continuous and monotonous stimulation. In this study, we analyzed the potential clinical relevance of the ASSR adaptation. ASSR were elicited in eight anesthetized adult rats by 8-kHz tones, modulated in amplitude at 115 Hz. We called independent epochs to those EEG epochs acquired with sufficiently long inter-stimulus interval, so the ASSR contained in any given epoch is not affected by the previous stimulation. We tested whether the detection of ASSRs is improved when the response is computed by averaging independent EEG epochs, containing only unadapted auditory responses. The improvements in the ASSR detection obtained with standard, weighted and sorted averaging were compared. In the absence of artifacts, when the ASSR was elicited by continuous acoustic stimulation, the computation of the ASSR amplitude relied upon the averaging method. While the adaptive behavior of the ASSR was still evident after the weighting of epochs, the sorted averaging resulted in under-estimations of the ASSR amplitude. In the absence of artifacts, the ASSR amplitudes computed by averaging independent epochs did not depend on the averaging procedure. Averaging independent epochs resulted in higher ASSR amplitudes and halved the number of EEG epochs needed to be acquired to achieve the maximum detection rate of the ASSR. Acquisition protocols based on averaging independent EEG epochs, in combination with appropriate averaging methods for artifact reduction might contribute to develop more accurate hearing assessments based on ASSRs.

## Introduction

Auditory steady-state responses (ASSRs) are brain oscillations locked to the periodic properties of acoustic stimuli [1, 2]. Audiological tests based on the acquisition of ASSR are useful for estimating the hearing sensitivity, mainly because multiple hearing frequencies can be simultaneously assessed, and the auditory response can be objectively detected using statistical tests [3–7].

Typically, ASSR are elicited by the continuous presentation of amplitude modulated (AM) tones. The extraction of the auditory response from the measured signal essentially relies on averaging epochs of the EEG, time-locked to the stimulus [8]. Such a manipulation assumes that the EEG signal is a linear superposition of the highly stereotyped, time-invariant response, and the ongoing background noise [9]. However, evidence obtained in several sensory pathways suggest that the evoked potential amplitude might not be steady but decreases exponentially due to the serial and regular stimulation [10–12]. Such effect has been defined as evoked potential adaptation.

Evidence supporting the adaptation of auditory evoked potentials (AEP) has been provided by analyzing the effect of the stimulation rate on the amplitude and latency of transient responses. Those studies show that, as the presentation rate of acoustic stimuli increases, the amplitude of AEPs obtained in both humans and rodents decline [13–19]. When the time-course of transient AEPs amplitude has been analyzed, it has been observed that the asymptotic amplitude of the response is preceded by an initial stage, in which the amplitude decreases over several stimulations [10, 13, 20, 21].

Traditionally, it has been argued that the ASSRs primarily result from the linear superposition of transient AEPs elicited by the high presentation rate of acoustic stimuli [22, 23]. Nevertheless, unlike the suppression of transient AEPs induced by the stimulus repetition, the adaptation of ASSRs has received relatively little attention.

Several studies have analyzed the time course of the ASSR amplitude, describing the changes in amplitude resulting from the time-domain averaging of sequentially acquired EEG epochs, i.e., averaging epochs containing auditory responses elicited by continuous acoustic stimuli [24–26]. They have systematically demonstrated that the amplitude of the ASSR gradually decreases as the first EEG epochs are averaged and remains stable when subsequent epochs are included in the time-domain averaging. in those studies, the amplitude of the auditory response has been assumed as stationary. Consequently, their results have been explained by the relatively high contribution of the un-averaged noise to the response amplitude computed in the first epochs of the recording, which is attenuated as averaging is performed [5, 27–29].

The estimation of the ASSR during the time-domain averaging of epochs is a cumulative process, in which the response amplitude computed from a given epoch relies on those obtained in the preceding EEG segments. Consequently, the ASSR computed at a given time after the stimulus onset might not necessarily correspond to the instantaneous ASSR amplitude -as subsequent epochs of a recording cannot be considered statistically independent. Therefore, the standard time-domain averaging of subsequently acquired epochs does not allow to differentiate between methodological and physiological related variations in the amplitude of the ASSR [30]. For assessing the adaptation of the evoked potentials, it is necessary that the response amplitude estimated at a given time window is not compromised by those computed in the preceding EEG segments. In other words, the ASSR amplitudes should be extracted without epochs being time-domain averaged with the preceding EEG segments. In that scenario, similar ASSR amplitudes over time would strongly support the strict stationary behavior of the ASSR. Alternatively, an exponential decrease of amplitude as a function of time would accounts for the adaptation of the response.

Since computing the amplitude of evoked potentials at the level of single trials might be controversial, we analyze the stability of ASSR using the traditional methodology proposed by Ritter et al. [13] for quantifying the adaptation of cortical AEP. In summary, such methodology consists of acquiring several recordings of the same experimental condition. Following, instead of averaging subsequent epochs within the recordings, the “instantaneous” ASSR amplitudes are computed by averaging those epochs which correspond to the same time window in the different recordings. Using such methodology, we have demonstrated that the amplitude of ASSRs generated in the brainstem decrease exponentially due to the sustained presentation of AM sounds [30]. That behavior might reflect the loss of novelty of the sensory input, increasing the sensitivity to relevant fluctuations in the acoustic environment [13, 31].

The adaptation of ASSR might have implications in the clinical practice, especially when recording protocols are based on averaging a reduced number of sequentially acquired epochs [6, 28, 29]. In such a practical situation, the ASSR computed after the completion of the averaging might be strongly influenced by the adaptation of the response. This is important considering that the ASSR amplitude estimated at the end of the recording is used to judge the significance (statistical detection) of the auditory responses.

Possible shortcomings in the computation of ASSRs resulting from adaptation might be overcome by implementing stimulation protocols which prevent the suppression of the ASSR amplitude over time. In practice, the acquisition of unadapted ASSR can be achieved by replacing the continuous acoustic presentation of tones commonly used to elicit ASSRs by a discrete presentation mode -in which segments of AM-sounds of a few seconds in length are presented with a given inter stimulus interval (ISI). Using an adequately long ISI would imply that consecutive epochs can be considered statistically independent events, i.e., they are different, independent measures of the same variable. From a physiological point of view, this means that the auditory response embedded in any unaveraged epoch is not affected by the preceding stimulation. In such a situation, the neural population synchronously responding to the incoming stimulus would be equal or only slightly different in size compared to the number of neurons that responded to the preceding stimulation [17, 32]. Consequently, the amplitude of the auditory response would remain relatively steady across trials. Based on the physiological processes mentioned above, we will call independent epochs those EEG epochs acquired with sufficiently long inter-stimulus interval, so the ASSR contained in any given epoch is not affected by the previous stimulation.

Additionally, a better estimation of the response might result from the implementation of averaging procedures that attenuate the effect of motion and muscular artifacts, i.e., using weighted and sorted averaging methods [33–35]. Weighted averaging involves normalizing the voltage samples of each individual EEG epoch by an estimate of the amplitude variability, e.g., weighting the data samples by the inverse of either the variance, or the standard deviation of the voltage amplitude [33]. Sorted averaging comprises the rearrangement of epochs as a function of the voltage variability, averaging only those epochs which contribute to increasing the accuracy of the response estimation. The latter is typically achieved by sorting the epochs in an ascending order of their root-mean-square (RMS) and averaging first those epochs with low RMS, as they contribute to increasing the signal-to-noise ratio (SNR) of the measurement [36, 37]. Both weighted and sorted averaging have been applied to the analysis of ASSR. Weighted averaging is already available in commercial ASSR systems, and it is commonly used for research purposes [6, 25, 38]. Sorted averaging has only been tested experimentally [39], probably due to the relatively high computational cost of storing and sorting a large number of epochs during the online estimation of ASSRs.

As mentioned above, we have previously demonstrated that the ASSR generated in the brainstem of rats adapts to the sustained stimulation [30]. However, a further quantification of the ASSR adaptation is needed to determine its possible relevance and implications for hearing assessments. In this regard, it is important to note that a significant decrease of the ASSR amplitude over time has been also obtained when analyzing the stability of cortical ASSRs in humans [40]. However, based on the small differences between the initial ASSR amplitude and that obtained after 92-s of stimulation, those authors concluded that such a decline in amplitude is not relevant for the clinical practice. Therefore, in this study, we analyzed possible biases in the computation of the brainstem ASSR resulting from adaptation. More specifically, we tested whether the detection of ASSRs generated in the rat brainstem is significantly improved when the response amplitude is computed by averaging statistically independent EEG epochs containing only unadapted auditory responses.

On the other hand, both weighted and sorted averaging are artifact reduction protocols which have been implemented considering stationary auditory responses. Therefore, the efficacy of those algorithms needs to be re-evaluated in scenarios in which changes in SNR are associated not only with noise variability, but also with the dynamics of the response. Consequently, we analyzed the validity of weighted and sorted averaging for detecting adaptive responses, and the improvement in the ASSR detection resulting from averaging statistically independent epochs as a function of the averaging protocol. We discussed the results based on a comparison with existing paradigms designed to optimize the detection of ASSR. Additionally, we addressed the discrepancies between the brainstem ASSR adaptation obtained in rats [30] and the lack of adaptation of the human 40-Hz ASSR reported by Van Eeckhoutte et al. [40].

## Materials and methods

### Experimental subjects

Auditory responses were obtained from 8 adult Wistar rats. Animals were housed in a standard bio-clean animal room under a 12-h light-dark cycle at 22–24°C, with free access to food and tap water. To perform the recordings, animals were anesthetized with ketamine (75.0 mg/kg, ip) and diazepam (5.0 mg/kg, ip). Supplemental doses of anesthesia were administered during the experiment at a level sufficient to maintain the animal in an areflexic state. Atropine sulfate (0.06 mg/kg; im) was administered to decrease the mucosal secretions. Body temperature was maintained at 37.0±0.1°C by a body temperature control system (Bioseb, model LE-6400). Due to the experimental procedure, the sacrifice of the animals was not necessary. They were returned to the colony after recovering from anesthesia. The present study was performed under approval of the Animal Research and Ethics Committee of the Cuban Neuroscience Center, conformed to the guidelines of the National Center for Animal Breeding of Cuba.

### Acoustic stimuli

Continuous tones of 8 kHz sinusoidally-modulated in amplitude (95% depth) at 115 Hz were generated using the ASSR software module [41] of the AUDIX system (Havana, Cuba) and presented monaurally at 50 dB SPL, via an ER 3A Etymotic Research insert earphone. Custom-fitted ear molds were used to replace the original foam to permit the earphone to be coupled to the rat’s ear. Acoustic stimuli of 8 kHz have been previously used to study the ASSR in rats [42–44], since this frequency corresponds to a peak in the spectral hearing sensitivity of rats [45, 46]. The acoustic levels are referred to a Brüel & Kjær artificial ear (type 4152). Calibration was performed using a Brüel & Kjær 2250 sound level meter (Brüel & Kjær 4144 microphone).

### Recordings

Electrophysiological responses were recorded differentially using stainless-steel needle electrodes inserted subdermally (vertex positive; neck negative; thorax reference). Recordings were amplified with gain 1.2x10^4^ and band-pass filtered–cutoff frequencies of 10 and 300 Hz. Output of the filter was digitized at 16 bits of resolution and sampled at 920 Hz. Segments with peaks of electrical oscillations exceeding 50 mV were rejected online. Typically, less than five segments per recording were rejected and they were randomly distributed across epochs. Data acquisition continued until completing 60 artifact-free epochs of 4.45 s (4096 time-points each). Thirty recordings were acquired from each animal. During the experimental session, every recording was preceded by a no-stimulation period of ten minutes.

### Pre-averaging modifications of epochs

**Fig 1:**
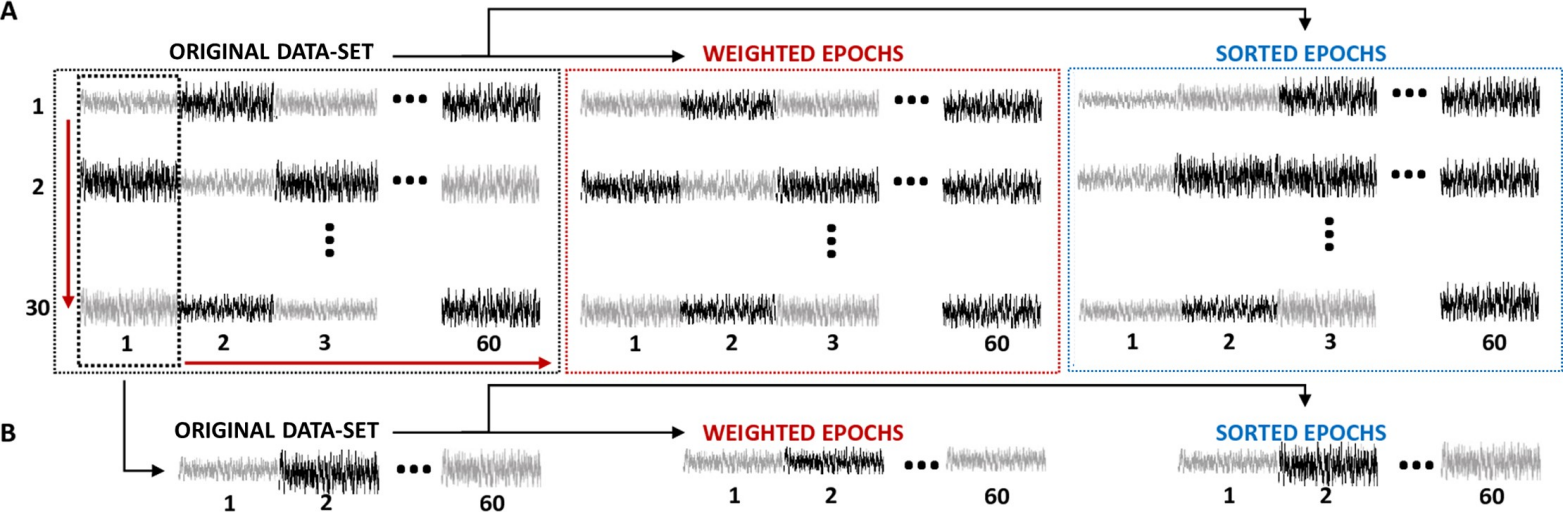
Diagram illustrating the arrangement of measured EEG epochs as a data matrix with 30 rows (recordings) and 60 columns (epochs within recordings). A) Epochs in the original dataset were weighted by the inverse of its variance and adequately normalized, resulting in epochs with equivalent amplitude variability (middle matrix). In addition, epochs in the original data set were sorted in every row, following an ascending order of RMS (right matrix). B) Epochs corresponding to the first time window in the different recordings were concatenated to obtain a new synthetic recording. Weighting and sorting of epochs forming the original synthetic recordings was performed. Note that, the amplitude variability among epochs is reduced by the weighting procedure. As a consequence of the sorting, epoch with lower amplitude variability (lower RMS), are located at the beginning of the recording.

Processing of the data was performed using in-house Matlab codes (MathWorks, USA). The 60 sequential epochs of the 30 recordings were re-arranged offline into a data matrix of 30 rows (recordings) and 60 columns (epochs within recordings) (Fig 1, left panel). From this dataset we created two other modified data matrices, containing weighted and sorted epochs, respectively (Fig 1A, middle and right panels). Both manipulations are based on assuming that the sample variability of the epoch reflects only the contribution of noise, so that noisy epochs will have higher amplitude variability [33, 36].

Weighted epochs were obtained by dividing each voltage sample by the amplitude variance of the epoch they belong to, so that variance was used as a measure of amplitude variability and weighting factor [25]. Pertinently, we normalized the weights by their average across all epochs in order to make the ASSR amplitudes obtained with the three averaging procedures comparable. In the sorting procedure, epochs were rearranged following an ascending order of root-mean-square (RMS). This parameter has been used before as sorting factor in the detection of ASSRs, as it is assumed to be proportional to the level of background noise [39]. In our experimental data, we confirmed that there were not preferential locations of the epochs after the sorting procedure. Since the sorting was performed separately for each recording, such manipulation implied that epochs corresponding to the same time window in different rows of the original matrix, will likely appear in different time windows of the ordered dataset.

### Adaptation of ASSRs

The adaptation of the ASSR was analyzed as described in Prado-Gutierrez et al. [30]. In summary, epochs in the data matrices were column-wise averaged (Fig 1A). In other words, the 30 epochs corresponding to the same time window in the different recordings were averaged in the time-domain. The column-wise averaging of epochs was performed for each dataset (original, weighted and sorted epochs). Noteworthy, for the sorted dataset, sorting was performed according to the RMS of epochs within each row, while the averaging was carried out column-wise, thus averaging epochs that did not correspond to the same time window in the original dataset.

The amplitude of the ASSR was computed once for each column, at the end of the averaging, using the fast Fourier transform (FFT). We used an FFT length of 4096 time points, which corresponded to the length of an epoch (4.45 s) and led to a spectral resolution of 0.22 Hz. A windowing technique was not implemented. The amplitude of the ASSR was defined as the spectral amplitude obtained at 115 Hz (frequency of the amplitude modulations of the acoustic stimuli). The amplitude of the 30 FFT bins at each side of the frequency of the auditory response were vector averaged to calculate the residual noise level (RNL). The selection of such spectral region for the computation of the RNL is based on the relation between the amplitude and the frequency of the EEG. It is well-known that the spectrum of EEG background oscillations (o resting EEG) is characterized by a non-linear decrease with increasing frequencies, such that higher amplitudes are found for lower frequencies. Therefore, computing the RNL in spectral regions much lower than that of the ASSR might led to overestimate the RNL, making the statistical detection of the ASSR more conservative [47, 48]. The high frequency-specificity of the steady state response ensure that its amplitude is independent of those background oscillations with similar frequencies, which distributes fairly uniformly in that frequency region [24, 47, 48]. At present, the selection of a frequency band around that of the ASSR for assessing the RNL, is the procedure of choice in most of the ASSR detection protocols used for both research and clinical purposes. The spectral components of the noise corresponded to the frequency range between 108.3 Hz and 121.7 Hz.

The column-wise averaging of epochs removes the background noise in every column separately, making the ASSR amplitudes comparable to those obtained with the classical procedure of averaging many epochs within a recording. Such manipulation allowed to reliable compute “instantaneous”, not accumulative ASSR amplitudes, since subsequently acquired epochs corresponding to the same recording were not averaged. Therefore, plotting such “instantaneous” ASSR amplitudes as a function of column index, i.e., the number of the acquired epoch, allowed us to explore the evolution of the ASSR amplitude during the stimulation period. It is worthy of note that such evolutions were represented in previous studies using the stimulation time as the abscise in graphs [30, 40]. However, as a consequence of the sorting procedure, the dynamics of the “instantaneous” ASSR amplitudes obtained from the sorted dataset do not necessarily correspond to the time course of the auditory response during the stimulation period. Therefore, instead of plotting the ASSR amplitude as a function of time, these dynamics were represented in this study using the epoch number as the abscise label in graphs.

One-way ANOVAs (p<0.05) and the corresponding post-hoc analyses (Tukey HDS test, p<0.05) were performed to analyze the changes of the “instantaneous” ASSR amplitude and the RNL in the stimulation period. Since the ASSR generated in the brainstem of rats completely adapts 30-s after the stimulus onset [30], the analysis of the ASSR evolution was restricted to the first 10 EEG epochs.

### Computation of ASSRs elicited by continuous stimulation: combined effect of adaptation and the averaging procedures

We analyzed the effect of the standard, weighted and sorted averaging on the ASSR computed by using the classical method of averaging subsequently acquired epochs, when the ASSR was elicited by continuous stimulation (within recordings, in the different datasets). The direction of the averaging is represented by the horizontal line in the left panel (Fig 1A). The ASSR amplitude was computed after including each additional EEG epoch in the averaging. Therefore, subsequent amplitude values were computed by using the same epochs but one, which emphasizes the strong relation between each ASSR amplitude and those computed in preceding epochs.

The FFT parameters used for computing the ASSR within recordings were the same used for the computation of the response amplitude during the column-wise averaging of epochs, described above. Similarly, the ASSR amplitude and the RNL were defined as the spectral amplitude obtained at 115 Hz and the average of the spectral amplitude computed in the adjacent 30 frequency bins on both sides of the response, respectively.

According to the adaptive behavior, the ASSR amplitudes embedded in the first unaveraged epochs of a recording (unadapted responses) will be higher than those embedded in later unaveraged epochs (adapted responses). Therefore, it might be expected that the ASSR amplitudes computed with the classical sequential averaging of epochs within a recording (row-wise averaging) will vary as epochs with unadapted responses are included or not in the averaging. Therefore, we analyzed the evolution of the ASSR amplitudes during the averaging of a fixed number of epochs corresponding to the same recording (2, 4 and 8 consecutive epochs), when the first 1, 2, 4, 8, 16, and 32 epochs of the recordings were excluded from the averaging. The exclusion (rejection) procedure consisted in starting the averaging not from the first epoch of the recording (first element in the rows) but a given number of epochs after (from the row element 1+n, where n = 1, 2, 4, 8, 16 and 32). The rejection of epochs was performed in the original, weighted and sorted datasets.

A three-way ANOVA (p<0.05) and the corresponding post-hoc analyses (Tukey HDS test, p<0.05) were conducted to compare the ASSR amplitudes obtained at the end of the averaging, using as factors the averaging method, the number of averaged epochs, and the number of rejected epochs (number of epochs acquired at the beginning of the recording that were not included in the averaging). In the experimental design, the factor “averaging method” had three levels: standard, weighted, and sorted; the factor “number of averaged epochs” had: three levels: 2, 4, and 8 epochs; and the factor “number of rejected epochs” had seven levels: 0, 1, 2, 4, 8, 16, and 32 epochs.

### Unadapted ASSR computed by averaging independent EEG epochs

One of the main aims of this study is testing the conditions in which the averaging of independent EEG epochs (those acquired after a sufficiently long inter-stimulus interval, ISI) results in higher ASSR amplitudes than those obtained by averaging a combination of epochs containing unadaptated and adapted auditory responses. To this end, the first epochs in each recording (first column in the original dataset, containing unadapted responses) were concatenated to form a synthetic recording (Fig 1B). As mentioned before, the auditory response embedded in any of those epochs are not affected by the preceding stimulation, since recordings were obtained after ten minutes of resting. From the original synthetic recordings, weighting and sorting of epochs were then performed to finally prepare three types of synthetic recordings (Fig 1B). Epochs corresponding to the same dataset were sequentially averaged in the time domain. The ASSR amplitude was computed after including each additional EEG epoch in the averaging to construct its evolution with respect to the number of averaged epochs. It is important to note that this is the same procedure described in the for the row-wise averaging of epochs within the original recordings. Nevertheless, the synthetic recordings were formed by epochs without any direct temporal relationship between them. Therefore, a valid comparison between the evolution of the ASSR amplitude during the sequential averaging of original and synthetic recordings can be only performed if the ASSR amplitudes are plotted as a function of the number of averaged epochs.

For comparative purpose, the evolution of ASSR amplitude associated with the averaging of independent epochs in the synthetic recordings were contrasted with that obtained analyzing the first of the 30 original (first row in the datasets). This selection allowed us using the first epochs of the original recording also as the first epochs of the synthetic recording. Thereby, the initial ASSR amplitude computed from both types of recording using standard and weighted averaging were equalized. We performed that comparison separately for each of the three averaging methods (standard, weighted and sorted averaging). In each case, a two-way ANOVA (p<0.05) and the corresponding post hoc analysis (Tukey HSD test, p<0.05) was performed, using as factors the type of epoch (two levels: original data vs. synthetic data only containing unadapted responses) and the number of averaged epochs (10 levels: 1, 2, 3, 4, 5, 6, 7, 8, 9, and 10 epochs).

### Statistical detection of ASSRs

The ASSR amplitudes obtained by the classical sequential averaging of epochs within a recording (first row in the datasets) and those obtained by the sequential averaging of epochs within the synthetic recordings (formed by independent epochs containing only unadapted responses) were subject to statistical detection. For this end, the ASSR computed after averaging a given number of epochs was compared with the corresponding RNL, using the Hotelling's T2 multivariate test, which considers both the amplitude and phase of the oscillations [3]. The statistical test was applied after averaging each additional EEG epoch. ASSRs were considered as detected when the response amplitude was significantly higher than the RNL (one sample T2-test, df = [2,58], p<0.05). Detection rates were computed as the fraction of animals where the response was statistically significant.

## Results

### Quantification of the ASSR adaptation depends on the averaging procedure

**Fig 2:**
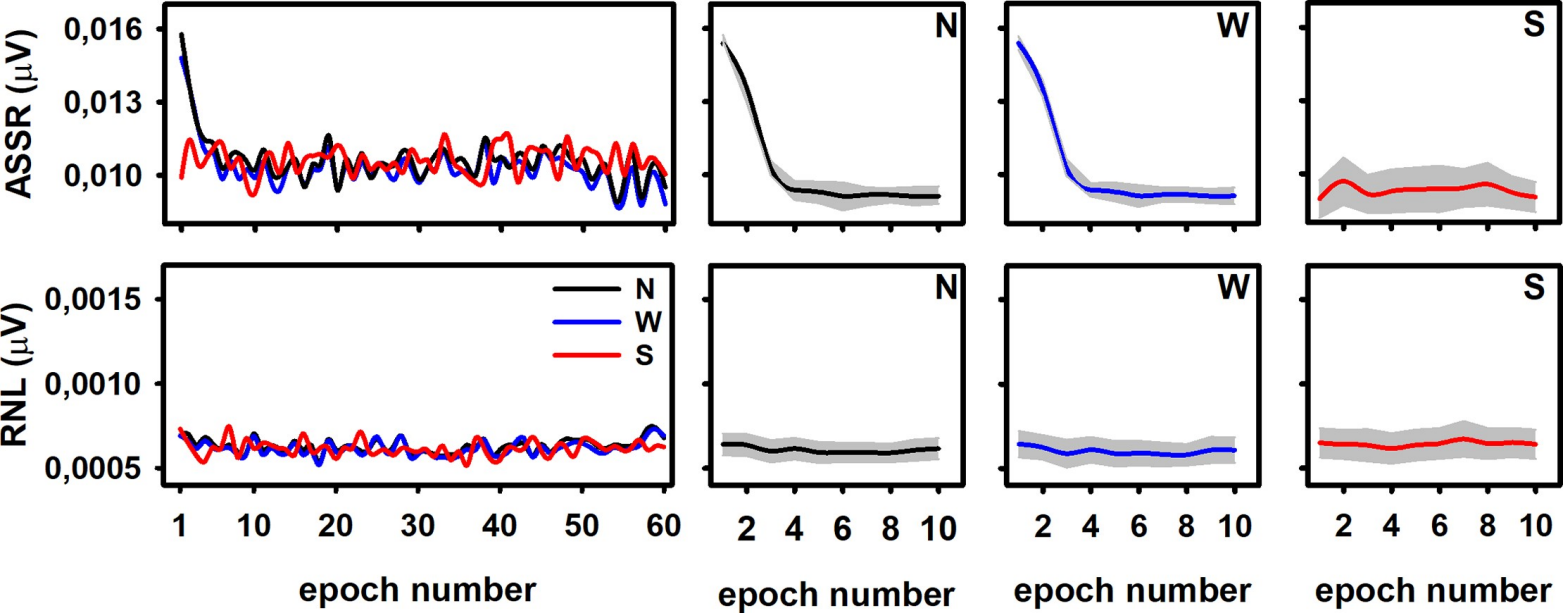
Evolution of the “instantaneous”, not accumulative ASSR amplitude (upper panels) during the stimulation period. The behavior of RNL (inferior panels) are also represented. Both the ASSR and the RNL are plotted as a function of the epoch number (index of the columns in the data matrices). Traces corresponding to a typical individual are displayed in the left panels. Means and standard deviations (8 individuals) are plotted in the rest of the panels for original (N), weighted (W) and sorted (S) datasets.

The upper panels in Fig 2 illustrates the ASSR amplitudes calculated after the column-wise averaging of epochs as a function of the location of epochs within the recordings -i.e., the epoch number, which represents the index of the columns in the data matrix. Since the averaging of epochs within recordings (row-wise averaging) was not performed, those traces do not represent the effect of adding more epochs to the averaging but the evolution of the “instantaneous”, not accumulative ASSR amplitude during the stimulation period. For the ASSR amplitudes resulting from the original and weighted datasets, such dynamics can be represented as a function of time, as previously shown in Prado-Gutierrez et al. [30]. However, as mentioned in the Method section, the stimulation time is not appropriate for representing the evolution of the ASSR amplitudes computed from the sorted dataset, as sorting implies changes in the time location of epochs for each recording. This is the reason why the evolutions in Fig 2 are represented as the variation of the ASSR amplitudes as a function of the epoch number -i.e., the index of columns in the datasets.

The adaptation of the ASSR was evident by analyzing the original and weighted datasets. In both cases, the ASSR amplitudes decreased over the first four EEG epochs and remained steady afterward (One-way ANOVA: F = 17.30, p<0.05 and F = 14.66, p<0.05 for original and weighted epochs, respectively). However, the adaptive behavior of the ASSR was not detected in the sorted dataset (One-way ANOVA: F = 0.88, p>0.05). As expected, the RNL resulting from the column-wise averaging of epochs was similar in the original, weighted and sorted data-sets. In all cases, the RNL did not vary as a function of the location of epochs within the recording, i.e., the RNL was constant among columns (Fig 2, lower panels) (F = 0.89, p>0.05, F = 0.93, p>0.05 and F = 0.25, p>0.05 for original, weighted and sorted datasets, respectively).

### The ASSR amplitude computed by averaging subsequent epochs within a recording is affected by adaptation

**Fig 3:**
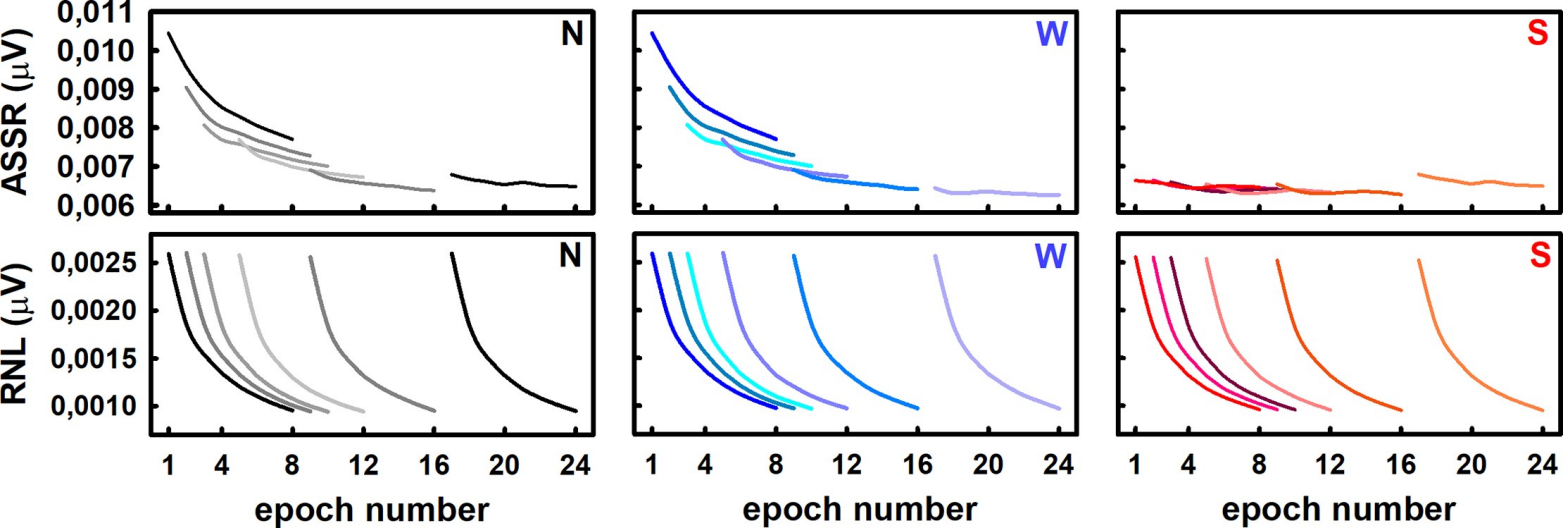
Evolution of the ASSR amplitude and the RNL during the standard (N), weighted (W) and sorted (S) averaging, when the ASSR is elicited by continuous stimulation. Traces represents the mean amplitudes of 30 recordings obtained in a representative individual. From left to right, traces in each chart correspond to the evolution of the ASSR amplitude during the sequential averaging procedure, when 0, 1, 2, 4, 8 or 16 epochs acquired at the beginning of the recordings were excluded from the averaging. For the sake of clarity, evolutions are represented with different colors (arbitrarily selected).

The time course of the ASSR obtained during the standard sequential averaging of eight epochs within a recording (row-wise averaging of epochs) is represented in Fig 3 (left upper panel). In that chart, traces represent the evolution of the ASSR amplitude (plotted as a function of epochs number) obtained when epochs containing unadapted auditory responses were included or not in averaging, i.e., the row-wise averaging might start from the first element of the row or from the element 1+n, where n = 1, 2, 4, 8 and 16. In all cases, the evolution of the ASSR were characterized by a progressive decrease of amplitude, which was mainly evident during the averaging of the first EEG segments (Fig 3, left upper panel).

Furthermore, the evolution of the ASSR during the standard averaging procedure depended on the number of epochs excluded from the averaging (Fig 3, left upper panel). As the number of rejected epochs increased, the ASSR amplitude of the first epoch included in the averaging decreased. Consequently, as the number of rejected epochs increased, the evolution of the ASSR during the averaging procedure was characterized by systematically lower decreases of amplitude. In fact, the ASSR amplitude decreased only slightly during the standard averaging procedure, when the first 16 epochs of the recording were rejected, i.e., when the row-wise averaging started in the 17^th^ element of the rows (black, right trace in the left, upper panel of Fig 3). Since the first elements of the rows in the original dataset are those epochs containing unadapted responses, the effect of the number of rejected epochs evolution of the ASSR during the standard averaging procedure can be interpreted as a direct consequence of the ASSR adaptation.

Similar behaviors resulted from applying weighted averaging (Fig 3, middle upper panel). Nevertheless, when epochs within recordings were sorted-averaged, the ASSR amplitude did not vary as the number of averaged epochs increased. Furthermore, the evolution of the ASSR amplitude during the sorting averaging did not vary as the number of rejected epochs increased.

**Fig 4:**
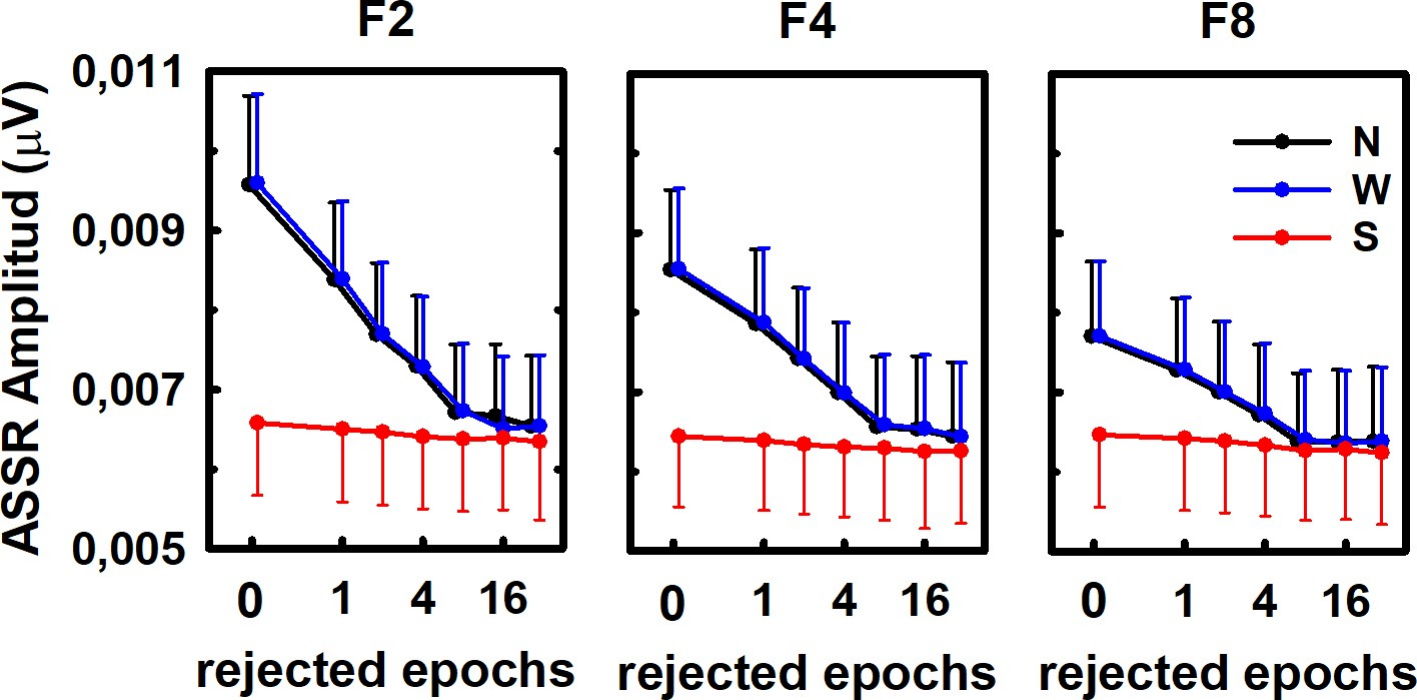
ASSR amplitudes as a function of the averaging method, the number of epochs and the number of epochs acquired at the beginning of the recording that were excluded from the averaging. F2, F4 and F8 represent the fixed number of epochs averaged before computing the ASSR amplitude (2, 4 and 8 epochs, respectively). Averaging protocols are also represented (N: standard, W: weighted, S: sorted). Plots represents the mean (symbol) ± standard deviation (vertical bar) across eight individuals. For the sake of clarity, the linear scale of the abscissa (x-axis) was modified.

Consequently, the ASSR amplitude obtained after averaging a given number of epochs within recordings depended on both the averaging method and the number of epochs acquired at the beginning of the recording that were excluded from the averaging (Fig 4; three-way factorial ANOVA: F = 5.66, p<0.05 and F = 3.20, p<0.05 for the effects of the averaging methods and the number of rejected epochs, respectively). The other factor in the statistical test (number of averaged epochs) did not have a significant effect on the ASSR amplitude (F = 1.56, p>0.05). Similarly, the interaction among factors did not any have significant effects on the ASSR amplitude (F = 0.60, p>0.05, for the interaction between averaging method and the number of rejected epochs; F = 0.14, p>0.05, for the interaction between the number of averaged epochs and the number of rejected epochs; F = 0.24, p>0.05 for the interaction between the number of averaged epochs and the averaging methods; and F = 0.03, p>0.05, for the interaction among the three factors).

The result of the post hoc test (Tukey HDS test) confirmed that, when using the standard and weighted averaging methods, the ASSR amplitude computed by averaging only two consecutive EEG epochs within a recording significantly decreased as the number of epochs excluded from the averaging increased up to eight (Fig 4, left panel). Further increases in the number of rejected epochs did not have a significant effect on the response amplitude (Fig 4, left panel). Once again, a different behavior resulted from applying sorted-averaging. When this method was implemented, the ASSR amplitude obtained after averaging two EEG epochs did not significantly vary as a function of the number of epochs rejected before starting the averaging (Fig 4, left panel). Furthermore, when the number of rejected epochs increased up to four, the sorting averaging resulted in significantly lower ASSR amplitudes as compared to those obtained with the standard and weighted averaging protocols (Fig 4, left panel). The same trends were obtained when the ASSR amplitude was estimated by averaging four and eight epochs (Fig 4, middle and right panels).

The changes in the ASSR described above could not be explained by the behavior of the RNL during the averaging of epochs within recordings. Increasing the number of excluded epochs from the averaging did not have any effect on the behavior of the RNL (Fig 3, bottom panels). Furthermore, the evolution of the RNL during the sequential averaging of epochs within the recordings did not change as a function of the averaging method.

### ASSR amplitudes computed by averaging independent epochs

**Fig 5:**
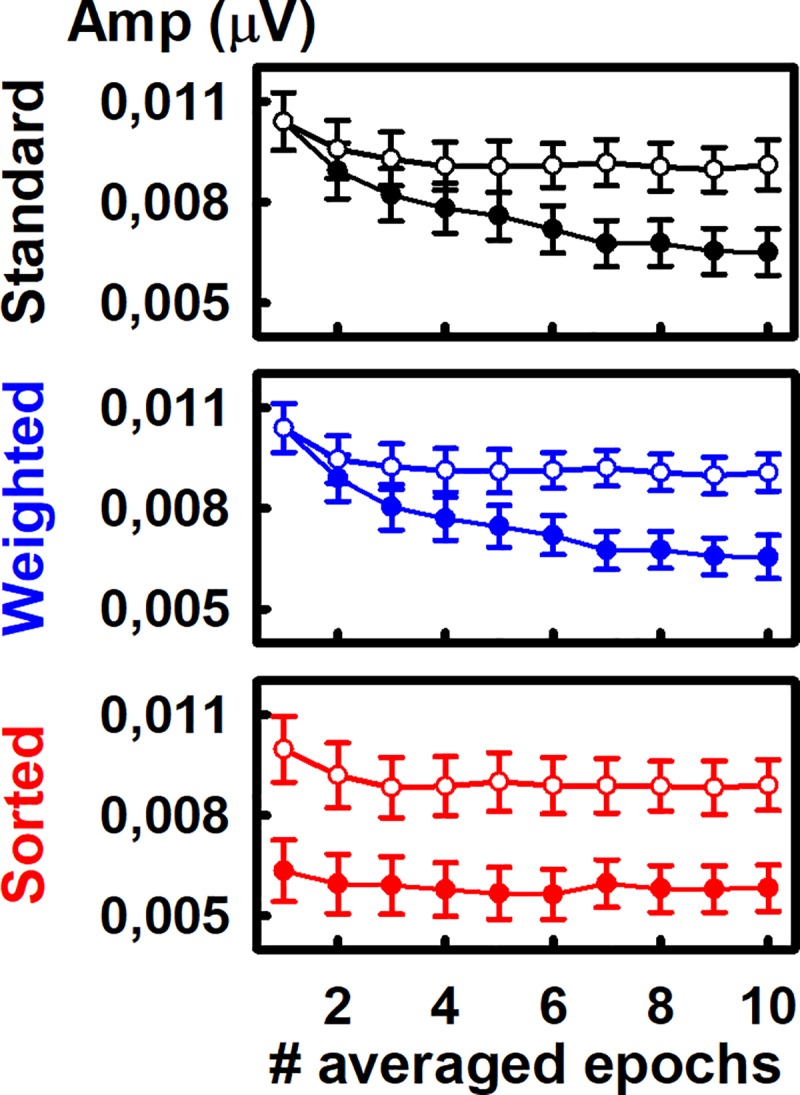
Evolution of the ASSR amplitude during the sequential averaging of EEG epochs containing unadapted responses (open circles, synthetic recordings) and the sequential averaging of epochs within the original recordings (filled circles). The latter corresponds to the classical procedure of averaging a combination of epochs containing unadapted and adapted responses. For each type of recording, the standard, weighted and sorted averaging are represented. Plots represent the mean ± standard error across 8 individuals.

Fig 5 illustrates the evolution of the ASSR amplitudes during the sequential averaging of epochs within the original recording (composed by epochs containing unadapteded responses and epochs containing adapted responses), and the behavior of the ASSR amplitude during the sequential averaging of epochs in the synthetic recordings (composed by independent epochs only containing unadapted auditory responses). By way of reminder, the synthetic recordings were constructed concatenating the first epoch of the 30 recordings acquired in each animal (as shown in Fig 1B).

When the ASSR were computed using standard averaging (Fig 5 upper panel), both factors “type of epoch” and “number of averaged epochs” had a significant effect on the response amplitude (two-way ANOVA: F = 42.60, p<0.05 and F = 5.38, p<0.05 for the effect of types of epoch and number of averaged epochs, respectively). The interaction between these factors did not have a significant effect on the ASSR amplitude (F = 1.24, p>0.05). Similar results were obtained when the ASSR was computed by weighted averaging (two-way ANOVA: F = 41.92, p<0.05; F = 5.01, p<0.05; and F = 1.10, p>0.05 for the effect of types of epoch, number of averaged epochs, and the interaction between these two factors, respectively). The result of the post hoc test (Tukey HDS test) confirmed that the benefit of computing ASSR amplitudes by the standard and weighted averaging of independent epochs was evident after averaging at least six EEG epochs.

When the ASSR amplitudes were computed using sorted averaging (Fig 5 lower panel), “type of epoch” was the only factor which had a significant effect on the ASSR amplitude (F = 143.98, p<0.05). Neither the number of averaged epochs nor the interaction between factors had a significant effect on the ASSR amplitude resulting from the sorted-averaging of epochs (F = 0.67, p>0.05; and F = 0.06, p>0.05 for the effect of type of epoch, number of averaged epochs, and the interaction between these two factors, respectively). The ASSR amplitudes computed by the sorted averaging of independent epochs were significantly higher than those computed by the sorted averaging of EEG epochs within original recordings. The difference in amplitude was evident even from the first epoch (Fig 5 lower panel). The ASSR amplitudes computed after sequentially averaging 10 independent epochs increased by 28.3%, 27,8% and 34.5% with respect to those resulting from the sequential averaging of epochs within the original recordings s, when the auditory response was computed using standard, weighted and sorted averaging, respectively (Fig 5).

**Fig 6:**
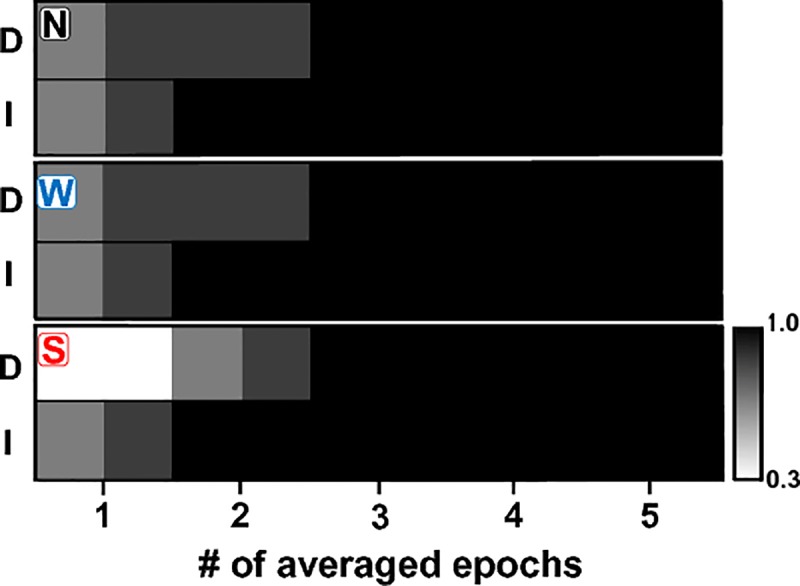
Detection rates of ASSR obtained by averaging a combination of epochs containing unadapted and adapted auditory responses (original recordings, D) and independent epochs only containing unadapted responses (I), as a function of the number of averaged epochs. Detection rates resulting from standard (N), weighted (W) and sorted (S) averaging are represented in the upper, middle and lower panels, respectively.

The changes in the ASSR resulting from averaging only independent epochs containing unadapted responses, instead of a combination of epochs containing unadapted or adapted responses, were reflected in the detection rate of the auditory response. As expected, when any of the three averaging methods were used, the detection rates of the ASSR increased as more epochs were averaged (Fig 6). When the ASSR amplitude was computed by standard and weighted averaging, the initial detection rates in both types of recordings were similar (Fig 6A and 6B). Nevertheless, the initial detection rate associated with the sorted averaging of independent epochs was 45% higher than that obtained by averaging sorted EEG epochs in the original recordings (Fig 6C). More importantly, computing the ASSR amplitude by averaging independent epochs, using any of the averaging procedures, halved the number of EEG segments needed to be averaged to achieve the maximum detection rate of the response.

## Discussion

In this work we tested the theoretical principles of an acquisition paradigm in which the detection of the ASSR is improved by reducing the adaptation of the ASSR. Our results demonstrate that, in the absence of EEG artifacts, the computation of the ASSR amplitude elicited by continuous stimulation vary as a function of the averaging method used in the acquisition procedure. When a continuous stimulation is delivered, the ASSR adaptation previously described in Prado-Gutierrez et al. [30] using the standard averaging of epochs is still present when the ASSR amplitudes are computed using weighted averaging. In the same conditions, sorted averaging may result in under-estimations of the ASSR amplitude. The effect of the averaging method is not evident when the ASSR amplitudes are computed by averaging independent EEG epochs containing unadapted ASSR, which in practice can be achieved by using a discrete stimulation mode instead a continuous presentation of AM-acoustic stimuli. Our results demonstrate that averaging independent EEG epochs containing unadapted auditory responses result in significantly higher ASSR amplitudes than those obtained by averaging a combination of epochs containing unadapted and adapted responses, depending on the averaging method and the number of averaged epochs used for the computation of the response. Consequently, averaging independent EEG epoch can significantly improve the detection of ASSRs.

### ASSR adaptation

The evolution the ASSR amplitudes described in Fig 2 (upper panels) replicates our previous findings and supports the notion that the ASSR adapts to the continuous presentation of acoustic stimuli [30]. These results are in accordance with studies describing the adaptation of the transient AEP in both humans and animal models [10, 13, 19–21, 49–51].

In this study we also provide a more precise description of the ASSR adaptation by using smaller FFT windows that than applied in our previous study. Noteworthy, we found new evidence of the ASSR adaptation by analyzing the dynamics of the ASSR during the stimulation period after the weighted and sorting of epochs within recordings.

On one hand, the ASSR adaptation is evident after the weighting procedure. This result is explained by the fact that we used the variance of each epoch as the weighting factor. Such parameter does not depend on the mean voltage value of the epoch. Moreover, due to the small amplitude of the auditory response relative to the background noise, the variance of the epoch mainly reflects the variance of the noise. As the variance of the noise seemed to be stable for all epochs, as partially reflected in analysis of the RNL displayed Fig 2, the weighting did not affect the evolution of the instantaneous, non-accumulative ASSR amplitude.

On the other hand, the ASSR amplitude resulting from the column-wise averaging of epochs in the sorted dataset was constant among columns (Fig 2). That result was a consequence of the small contribution of the auditory response to the RMS of single epochs when compared with the contribution of the background noise, even in those epochs containing unadapted ASSR. Due to the small contribution of the auditory response to the RMS, epochs containing unadapted ASSR were not placed in any preferential location of the recording after the sorting procedure. Consequently, any given column in the sorted dataset was mainly composed by epochs containing adapted ASSR. Therefore, the similar ASSR amplitudes obtained among columns are due to the relatively equal distribution of epochs containing unadapted ASSR among columns of the sorted dataset.

From a phenomenological perspective, the behavior of the ASSR described in this study meet the principal criteria defined by Thompson and Spencer [52] for adaptation: the exponential decrease of the response amplitude over time. As recently suggested by Duque et al. [19] analyzing auditory brainstem responses (ABR) of anaesthetized rodents, the adaptation of scalp recorded AEP reveals the adaptation of specific neural populations in the auditory pathway. However, in addition to adaptation, other physiological processes such as refractoriness might also contribute to the dynamics of the ASSR. As suggested by the experimental results and the theoretical model presented by Zacharias et al. [17], refractoriness might play a relevant role at periods of time shorter than 5 s. Therefore, it can be speculated that the balanced activation of a sub-pool of neurons which are refractory to the stimulation and another composed by neurons which are in a recovery-after-refractoriness stage, might contribute to explain the asymptotic amplitude of the ASSR.

### Cortical vs. brainstem ASSR adaptation

A recent study, using the methodology implemented in [30] for quantifying the ASSR adaptation, reported a significant but very weak decrease in the amplitude of the human 40-Hz ASSR over time, concluding that the 40-Hz ASSR does not adapt to the continuous stimulation [40]. Those authors accounted for the discrepancies with our results based on differences in the ASSR neural generators (cortical versus brainstem) and differences between species (humans versus rats).

The reduced adaptation of the cortical ASSR can be explained as part of the gradient in the levels of neural adaptation existing from the auditory periphery to the cortex [53, 54]. Such a gradient is reflected in the different adaptation pattern of the human ABR with respect to that of the auditory middle latency response (MLR) [55, 56]. It is also evident when analyzing the sensitivity to the inter-stimulus interval (ISI) on the earlier relative to the later components of the AEP in rats [18].

Nevertheless, it is important to note that the anatomical organization and the physiological mechanisms of the auditory system are very consistent across mammals. These homologies are reflected in several properties of AEP recorded from humans and rodents [57–59]. Remarkably, similarities between humans and rodents have been well documented when analyzing the suppression of cortical AEP as a function of the inter-stimulus interval [14, 18]. Parallels between auditory oscillatory responses of rodents and humans have also been reported [43, 44]. Testing the adaptation of the 80-Hz ASSR of humans is necessary for a possible validation of the results obtained in animal models. Nevertheless, in our opinion, the interspecific differences should not be decisive to explain the different results presented here (and previously reported in Prado-Gutierrez et al. [30]) and those obtained by Van Eeckhoutte et al. [40]. Instead of the interspecific differences, we would like to draw attention to the combination of methodological parameters used to compute the ASSR amplitude in these studies.

### Effect of the analysis parameters on the computation of the ASSR amplitude

The lack of adaptation of the human 40-Hz ASSR reported by Van Eeckhoutte et al. [40] was obtained using an FFT window of 20.48 s. Such an FFT window is long enough to mask the brainstem ASSR adaptation reported in this study (which occurs in the first 15–30 s of stimulation). Epochs of 20.48 s are two to four times longer than those used in previous studies analyzing the time course of the human 80-Hz ASSR [24–26]. Remarkably, they are also much longer that those used in basic researches on the human 40-Hz ASSRs, in which the auditory response has been estimated using FFT windows of up to four seconds. These studies include correlation analysis between the 40-Hz ASSR and behavioral thresholds [60], objective estimates of the loudness growth function based on ASSR [61], the consistency of the 40-Hz ASSR across sessions [62], and the desynchronization of phase-locked neural activities associated with binaural processing [63].

**Fig 7:**
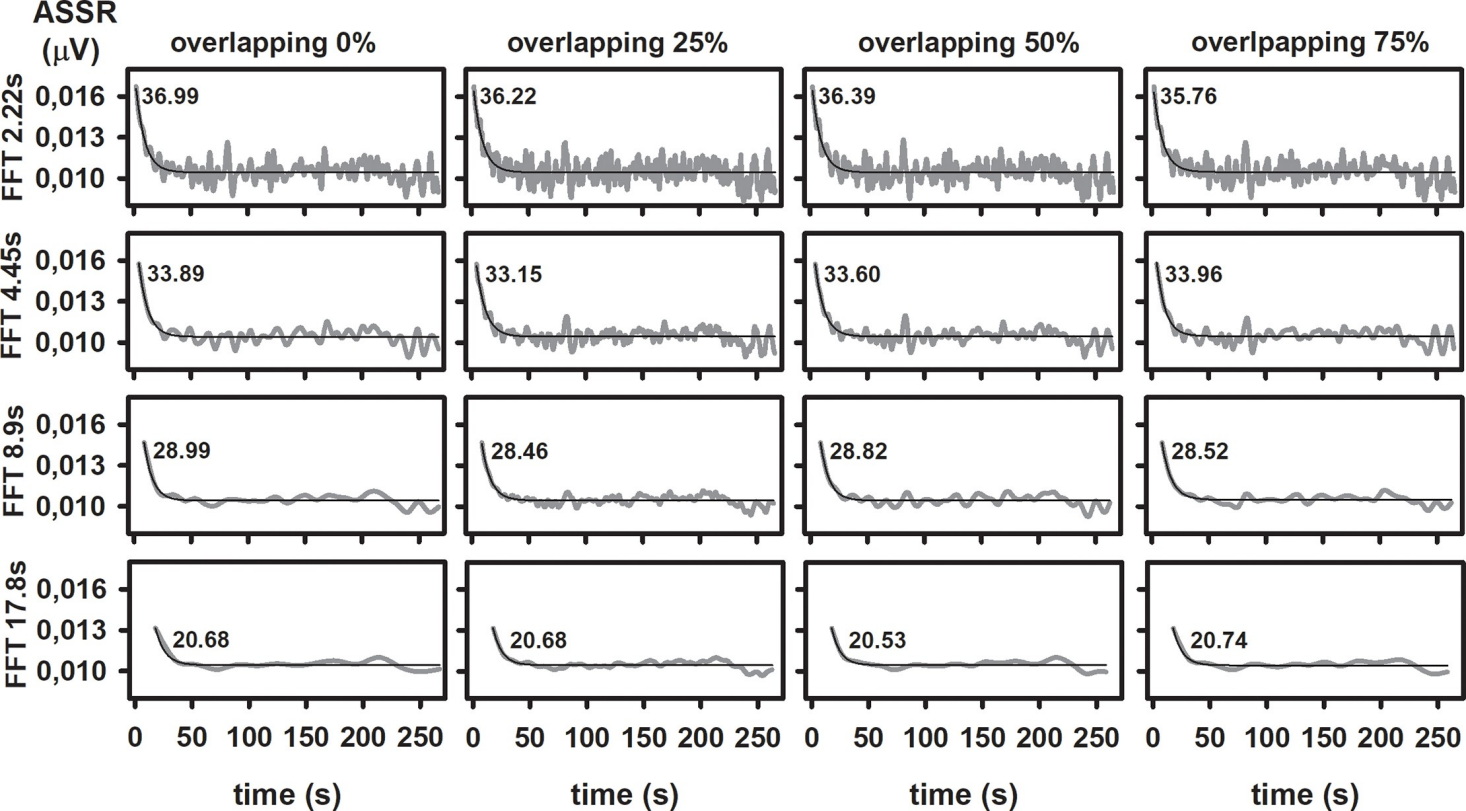
Effect of the epoch length (represented in y-label) and epoch overlap on the detection of the ASSR adaptation. The time evolution of the ASSR amplitudes (grey lines) were fitted to negative exponential functions (thin black line). Since using different length of FFT windows implies obtaining different number of measurements, ASSR amplitudes are plotted as a function of the recording time. The adaptation indexes are printed inside the corresponding charts.

Results presented in Fig 7 provides an example of how the detection of the ASSR adaptation can be influenced by the analysis parameters. They represent the evolution of the instantaneous, non-accumulative ASSR amplitude during the stimulation period, analyzed as a function of the epoch length and the overlapping of the FFT window used for the computation of the response. The procedure for obtaining such dynamics was the same used for analyzing the adaptation of the ASSR (see method section). More specifically, we performed the column-wise averaging of epochs of the original dataset, using different epoch lengths (2.22, 4.45, 8.9 and 17.8 s). Additionally, subsequent epochs within the recordings were partially overlapped using sliding windows. For a given epoch length, we tested 0, 25, 50 and 75% of overlapping. For the different combinations of these parameters, the ASSR amplitudes were computed once for each column, at the end of the column-wise averaging of epochs. The ASSR amplitudes were plotted as a function of the index of the columns in the dataset, which in this case is equivalent of plotting the ASSR amplitude as a function of the stimulation time. The time evolutions of ASSR amplitude were fitted to a decreasing exponential function. The adaptation index (*P*_*adapt*_) was calculated for each parameter combination, using the equation:

$$P_{adapt}=100({Amp}_{max}-{Amp}_{adapt})/{Amp}_{max}$$

Where *Amp*_*max*_ represents the maximum amplitude of the fitted curve and *Amp*_*adapt*_ represents its asymptotic value.

As shown in Fig 7, the length of the FFT window used for the computation of the response is critical in detecting adaptive behaviors (quantified here with the adaptation index). More specifically, the ASSR adaptation is progressively smeared as the length of the FFT window increases. Although greater overlapping implies greater time resolution, such a manipulation did not modify the adaptation index of the ASSR computed with a given FFT window. These exploratory results suggest that using FFT windows longer than 20 s will certainly mask the adaptation behavior of the ASSR in those cases in which the auditory responses is completely adapted in a shorter period. We consider that further studies should be carried out to rule out or confirm the adaptive behavior of the human 80-Hz ASSR.

### The computation of ASSRs elicited by continuous stimulation is affected by adaptation

After analyzing the error rate, the detection rate and the recording length of ASSR, Luts et al. [28] recommended using ASSR detection protocols with a fixed recording length and to judge the significance of the responses at the end of the recording. Furthermore, those authors noted that ASSR can be detected at the initial epochs of a recording, a result that was interpreted as false alarms caused by the greater influence of the background noise when there are few epochs in the averaging. Consequently, they suggest that a minimum of eight epochs should be averaged before computing the auditory response [28, 29].

Averaging a fixed number of EEG epochs (Fig 3), we corroborated the behavior of the background noise described in previous studies during the averaging of subsequent epochs within a recording [24, 25, 28, 29]. More importantly, we demonstrated that the amplitude of the ASSR is higher in the first epochs of the recording and that the evolution of the ASSR amplitude during the standard and weighted averaging of epochs rely upon the subset of epochs selected for the analysis. In other words, different dynamics are obtained whether the first epochs of the recording are included or not in averaging (Fig 3). That behavior is not supported by the evolution of the RNL, which is the same for all subsets of data. Therefore, our results indicate that the ASSR amplitudes computed during the averaging of epochs within a recording depends on whether epochs containing unadapted responses are considered or not in the averaging.

Consequently, the ASSR amplitude computed at the end of the averaging decreased as more epochs containing unadapted responses are excluded (Fig 4). From a practical point of view, these results highlight the need for defining not just an appropriate length of the recording and averaging stopping criteria for estimating ASSR amplitudes, but also when the computation of the response needs to be started.

### Is the discrete stimulation feasible?

The benefits of estimating the ASSR by averaging independent EEG epochs were evident when analyzing the amplitude and the detection rate of the response (Figs 5 and 6). As noted before, the acquisition of independent epochs for the computation of ASSR can be only achieved by using a discrete presentation of acoustic stimuli -in which segments of AM-tones of a few seconds in length are presented using a sufficiently long inter stimulus interval (ISI).

Due to the experimental design used in this work, we cannot make any statement about the minimum ISI required for enhancing the amplitude of the ASSR. Future experiments addressing that question are needed. Those studies should also focus on the effect of two other aspects of the stimulation strategy: the variability of the ISI and the presentation of broadband noise between consecutive stimuli. As described by Zacharias et al. [17], a semi-random presentation of acoustic stimuli around a mean ISI might reduce the predictivity of the stimulus, decreasing the magnitude of the response adaptation.

The result presented in Figs 5 and 6 also show that the amplitude and detection rate of ASSRs computed by averaging independent epochs did not vary as a function of the averaging method. In that regard, it is worth noting that our experiments were performed in anaesthetized animals, which were maintained areflexic along the recording session. Therefore, EEG artifacts were extremely uncommon. In this ideal scenario, it is expected that similar ASSR amplitudes will be computed by the weighted and sorted averaging of independent epochs. However, the tools for reducing the effect of EEG artifacts should be tested in scenarios closer to the clinical practice. In that respect, previous results suggest that the SNR obtained with sorted averaging is higher in comparison with that resulting from weighted averaging [39]. This advantage, combined with the fact that sorted averaging does not modify the amplitude of the auditory responses, makes this averaging method in a potentially powerful tool to improve the detection of ASSR.

A hypothetical issue regarding the feasibility of the discrete stimulation paradigm is the possible attenuation of the averaged ASSR amplitudes due to variations in the phase of the neural oscillations from one trial to another. Futures studies need to address this topic experimentally. Nevertheless, the ASSR phase estimated from independent epochs might be less variable than expected, as previous studies have reported a regularity in the expected phase of the human ASSR [29, 64, 65]. Similarly, the phase delay -a parameter related to the ASSR latency and that is calculated from the onset phase- has been consistent across studies [29, 65]. It is worth to note that, even if some phase-related attenuation of the ASSR might be present, our results suggest that applying a discrete stimulation might result in significantly higher ASSR amplitudes than those obtained using the conventional continuous stimulation mode.

In this study, ASSRs were evoked by AM-tones with standard sinusoidal envelopes. Modifications of the spectral composition and the amplitude envelopes of these standard stimuli have been proposed for optimizing the detection of ASSR [27, 66, 67]. Such modifications include the implementation of mixed amplitude and frequency modulated tones [68, 69], AM-tones with exponential envelopes [70, 71] and AM-noise [72]. Different physiological mechanisms underlie the increase in the ASSR amplitude resulting from the presentation of those “alternative” acoustic stimuli and that obtained by implementing a discrete stimulation mode. Since it is expected that the ASSR also adapts to the continuous presentation of “alternative” stimulation, further benefits in the detection of ASSR might be obtained when such stimuli are presented using the discrete stimulation mode proposed here. An ASSR acquisition protocol based on this stimulation paradigm in combination with appropriate averaging methods might lay the foundations for the development of more accurate hearing assessments based on ASSRs.
